# The long-term health outcomes, pathophysiological mechanisms and multidisciplinary management of long COVID

**DOI:** 10.1038/s41392-023-01640-z

**Published:** 2023-11-01

**Authors:** Jingwei Li, Yun Zhou, Jiechao Ma, Qin Zhang, Jun Shao, Shufan Liang, Yizhou Yu, Weimin Li, Chengdi Wang

**Affiliations:** 1 Department of Pulmonary and Critical Care Medicine, State Key Laboratory of Respiratory Health and Multimorbidity, Med-X Center for Manufacturing, Frontiers Science Center for Disease-related Molecular Network, West China Hospital, Sichuan University, Chengdu, China; 2 AI Lab, Deepwise Healthcare, Beijing, China; 3 Department of Postgraduate Student, West China Hospital, West China School of Medicine, Sichuan University, Chengdu, China; 4 Department of Computer Science, The University of Hong Kong, Hong Kong, China

**Keywords:** Infectious diseases, Immunological disorders

## Abstract

There have been hundreds of millions of cases of coronavirus disease 2019 (COVID-19), which is caused by severe acute respiratory syndrome coronavirus 2 (SARS-CoV-2). With the growing population of recovered patients, it is crucial to understand the long-term consequences of the disease and management strategies. Although COVID-19 was initially considered an acute respiratory illness, recent evidence suggests that manifestations including but not limited to those of the cardiovascular, respiratory, neuropsychiatric, gastrointestinal, reproductive, and musculoskeletal systems may persist long after the acute phase. These persistent manifestations, also referred to as long COVID, could impact all patients with COVID-19 across the full spectrum of illness severity. Herein, we comprehensively review the current literature on long COVID, highlighting its epidemiological understanding, the impact of vaccinations, organ-specific sequelae, pathophysiological mechanisms, and multidisciplinary management strategies. In addition, the impact of psychological and psychosomatic factors is also underscored. Despite these crucial findings on long COVID, the current diagnostic and therapeutic strategies based on previous experience and pilot studies remain inadequate, and well-designed clinical trials should be prioritized to validate existing hypotheses. Thus, we propose the primary challenges concerning biological knowledge gaps and efficient remedies as well as discuss the corresponding recommendations.

## Introduction

The coronavirus disease 2019 (COVID-19) pandemic has brought about an unprecedented scale of burden on health care systems.^1,2^ More than 770 million cases of COVID-19 and over 6.9 million fatalities have been documented since the outbreak.^3^ Severe acute respiratory syndrome coronavirus 2 (SARS-CoV-2), the causative agent of COVID-19, gains entry into host cells by combining with angiotensin-converting enzyme 2 (ACE2).^4,5^ Subsequently, SARS-CoV-2 undergoes replication and provokes damage to multiple organs/tissues, resulting in a complex array of clinical manifestations and potential long-term sequelae.^6,7^

**Fig. 1:**
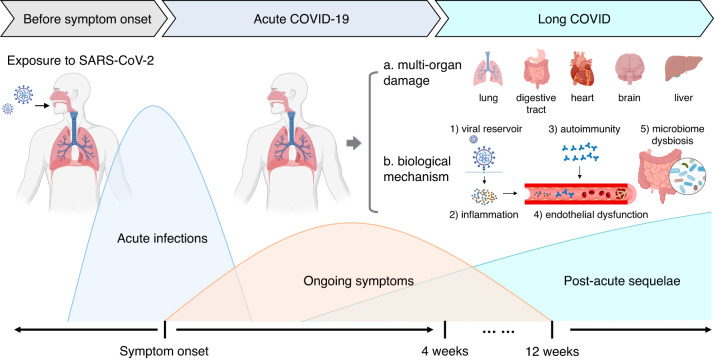
Timeline and multi-organ damage of long COVID. Long COVID is defined as the ongoing symptoms of COVID-19 patients between 4 and 12 weeks, or the post-acute syndrome over 12 weeks after the acute symptoms onset. In addition, the commonly involved organs and biological mechanisms are outlined

**Fig. 2:**
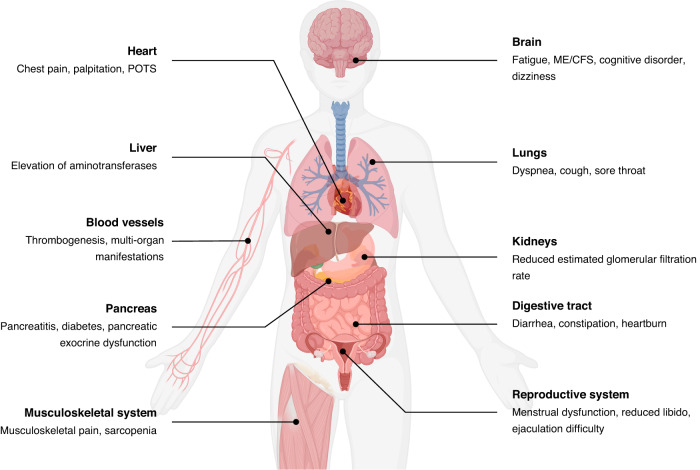
Multi-system symptoms/manifestations of long COVID. Long COVID has served as a multi-organ disease which can damage respiratory system, cardiovascular system, neuropsychic system, digestive system, circulatory system, musculoskeletal system, and genitourinary systems. ME/CFS myalgic encephalomyelitis/chronic fatigue syndrome, POTS postural orthostatic tachycardia syndrome

Long COVID, also referred to as ongoing symptomatic COVID-19 and post-acute sequelae of COVID-19 (PASC), is defined as symptoms of COVID-19 that persist for between 4 and 12 weeks or a post-acute syndrome at over 12 weeks after the onset of acute symptoms that cannot be attributed to any other illnesses^8–10^ (Fig. 1). According to recent research, patients with long COVID exhibit impairment of multiple organs, which manifests as a range of symptoms, including persistent fatigue, diarrhea, dyspnea, limited exercise tolerance, endocrine abnormalities, taste and smell dysfunction, and depression^11–16^ (Fig. 2). Furthermore, long COVID has been observed in a diverse spectrum of COVID-19 regardless of the mild or severe illness.^17–19^ Thus, it could be conceivably hypothesized that a more devastating effect could occur in the long COVID period than in the acute period of COVID-19. Nevertheless, the pathophysiological mechanisms and effective therapeutic choices regarding long COVID are unknown.

**Fig. 3:**
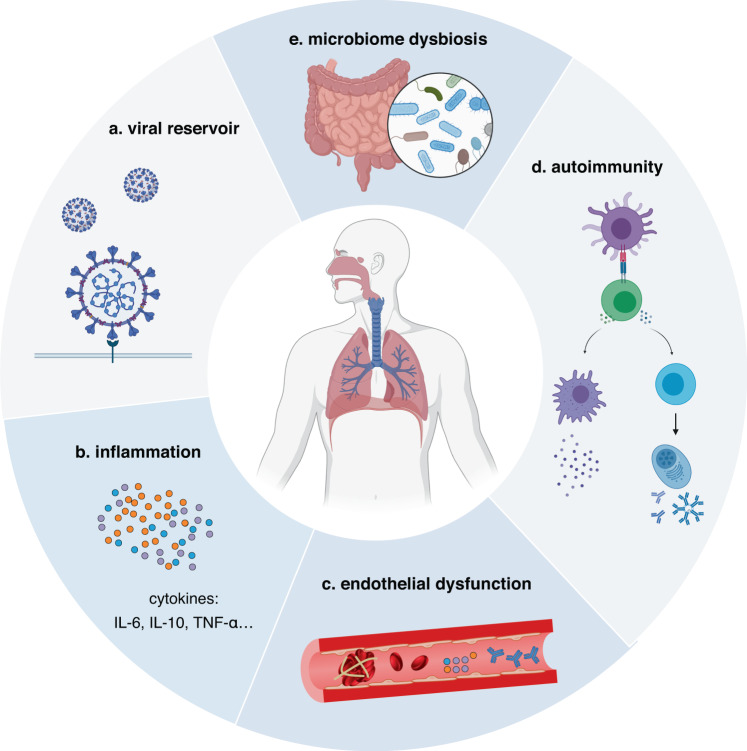
The potential pathophysiological mechanisms of long COVID. The main hypothesized pathophysiological mechanisms for long COVID include viral reservoir, gut microbiome dysbiosis, endothelial dysfunction, autoimmunity, and inflammation

Even so, multiple hypotheses concerning the pathophysiology of long COVID have been recently proposed (Fig. 3). The prevailing theories for underlying mechanisms comprise persisting viral reservoirs,^20^ sustained inflammation,^21^ host microbiome factors,^22,23^ persistent autoimmune responses,^24^ and endothelial dysfunction and subsequent blood clotting.^25^ Nevertheless, these studies regarding mechanistic hypotheses are mostly at the preliminary stage, and further research regarding the pathophysiology of long COVID is urgently needed.

Herein, we thoroughly describe the current understanding concerning the epidemiology, prevalent manifestations, pathophysiological mechanisms, and potential diagnostic tools and therapeutic options of long COVID. Furthermore, the present obstacles that need to be solved to advance long COVID research are discussed.

## Methods

To comprehensively understand the epidemiology, mechanisms, and management of long COVID, extensive literature searches were conducted of reputable databases, including PubMed, the Web of Science, EMBASE, and the Cochrane Library. The search spanned from January 2020 to June 2023, ensuring a thorough collection of relevant articles. The search terms involved “long COVID”, “post-acute sequelae of COVID-19”, “epidemiology”, “symptom”, “mechanism”, “management”, and their relative terms. Two authors screened the studies independently via the titles and abstracts. Afterwards, a meticulous review of the full texts of the eligible studies was performed. All included studies were written in English. Disagreements were reassessed by a third reviewer. Ultimately, the literature was read through and discussed by all authors to yield reliable conclusions.

### Epidemiology of long COVID

As the patient population recovering from acute COVID-19 continues to grow, recent studies have increasingly focused on investigating the post-acute effects of SARS-CoV-2 infection.^25–35^ An international observational study of individuals from 56 countries assessed the outcomes of 3762 confirmed or suspected COVID-19 patients within 6 months of infection via an online survey.^26^ The overwhelming majority of patients (65.2%) had at least one symptom at 6 months, of which fatigue (80%), post-exertional weakness (73.3%), and cognitive impairment (58.4%) were common clinical manifestations.^26^

Long COVID symptoms have also been recorded in studies conducted in China, providing significant data on the prevalence and characteristics of these symptoms. Long-term sequelae were evaluated in a cohort study comprising 1733 COVID-19 patients who had been discharged through the utilization of a range of assessments, including questionnaires, physical examinations, blood tests, a 6-minute walk test, ultrasonography, pulmonary function assessments, and imaging of the chest.^27^ At 6 months after the onset of symptoms, muscle weakness or fatigue was observed in 63% of cases, followed closely by sleep disorders (26%) and depression/anxiety (23%).^27^ In an identical cohort throughout a 2-year follow-up after acute SARS-CoV-2 infection, the morbidity rate of long COVID exhibited a substantial decline, dropping from 68% at 6 months to 55% after a 2-year period.^28^ Analogously, fatigue or muscle weakness was the prevailing manifestation, with an incidence of 30%.^28^ Sleep difficulties, hair loss, dizziness, and palpitations had a high incidence of over 10%.^28^ Although longitudinal improvements have been witnessed among studies, the prevalence of long COVID sequelae remains stubbornly high, implying that these sequelae may last long and lead to a tremendous burden on patients.

Studies from European countries have proclaimed similar results.^29–34^ In a French cohort with 150 confirmed COVID-19 patients, long COVID symptoms were collected via electronic medical records or telephone follow-up.^29^ At 60 days after symptom onset, 66% of patients had at least one sequela. Among these symptoms, the most frequent manifestation was asthenia (40%), followed by dyspnea (30%) and anosmia/ageusia (23%).^29^ According to a prospective cohort study from Spain, 50.9% of COVID-19 patients suffered from long COVID symptoms 10 to 14 weeks after illness onset.^30^ Furthermore, more detailed information regarding respiratory manifestations was assessed by spirometry or chest radiology. Reduced pulmonary function was noted in 9.3% of cases, and abnormal imaging results were found in 18.9% of patients.^30^ Analogously, data from the northern Netherlands involving 4231 COVID-19 patients and 8462 matched controls revealed that 21.4% of COVID-19 individuals and 8.7% of controls experienced persistent symptoms, including dyspnea, chest pain, painful muscles, lump in throat, ageusia/anosmia, heavy extremities, tingling extremities, paresthesia, and fatigue.^32^ In another study from the United Kingdom, a telephone screening instrument was designed to attain the post-acute COVID-19 sequelae and quality of life of patients with or without intensive care unit (ICU) care.^31^ Emerging COVID-19-related fatigue had the highest prevalence of 72% in ICU patients and 60.3% in non-ICU patients, and the alarming incidence of breathlessness as well as psychological distress was also emphasized.^31^

In an observational cohort study reporting 60-day outcomes from 38 hospitals in Michigan, the United States, medical records and telephone follow-up were adopted to aggregate longer-term consequences.^35^ After a 60-day period following discharge, 488 (41.8%) patients completed the follow-up, of whom 159 (32.6%) had persistent symptoms related to COVID-19, 75 (15.4%) had a sustained cough, 81 (16.6%) had wheezing/chest tightness/breathlessness, 44 (9.0%) experienced difficulty in ambulation, and 112 (23.0%) suffered from breathlessness when walking up stairs.^35^ In addition, according to the Morbidity and Mortality Weekly Report (MMWR) from the United States, 38.2% of participants with SARS-CoV-2 infection versus 16.0% of controls had long-term sequelae.^36^ Notably, the occurrence of long COVID was significantly more prevalent in individuals aged ≥65 years (45.4%) than in patients aged 18–64 years (35.4%).^36^ Thus, prevention strategies for long COVID sequelae should be emphasized, particularly in elderly individuals with COVID-19.

The aforementioned evidence has significantly enhanced our understanding of the manifestations of long COVID and aided in the identification of individuals at a heightened risk of developing sequelae following SARS-CoV-2 infection. Due to a number of factors, such as follow-up time, heterogeneity in self-reported data, variations in SARS-CoV-2 strains, and race and ethnicity, there are differences in long COVID epidemiology in distinct investigations. In addition, symptomatic differences are present in hospitalized and non-hospitalized individuals. Recent studies have indicated that participants allocated to general wards or ICUs had a higher likelihood of experiencing long COVID symptoms than individuals who were not hospitalized.^37,38^ However, hospitalization seems to be the only risk factor for complication probability rather than the severity of long COVID.^39^ These outcomes indicate that a large number of patients are likely to have long COVID, and efficient prevention and treatment strategies are warranted for combating numerous clinical manifestations. Moreover, the association between plentiful hazard factors and morbidity of long COVID remains to be investigated.

### Impact of variants and vaccines on long COVID

Since the COVID-19 pandemic, multiple variants have emerged with enhanced transmissibility, which may contribute to the increased number of patients with severe illness and even long COVID manifestations.^40–42^ The data from the Israeli nationwide health care organization revealed that long COVID symptoms associated with infections with all variants, including prototype, Alpha, and Delta variants, remained consistent.^43^ Nevertheless, another study revealed that infection with the prototype variant was associated with a higher prevalence of long COVID symptoms than infection with the Alpha or Delta variant.^44^ Among these variants, dyspnea was found to be more prevalent in patients infected with the prototype variant, while hair loss was more commonly observed in patients infected with the Delta variant.^44^ Interestingly, the prevalence of fatigue was found to be similar across both variants.^44^ Compared to these variants, the current data suggested that the Omicron variant possibly led to fewer clinical manifestations of long COVID.^45^ A study from Eastern India revealed that approximately 8.2% of patients infected with the Omicron variant self-reported experiencing long COVID manifestations, which is relatively lower than estimates for those infected with the Delta variant.^46^ Similarly, 4.5% of patients with an Omicron variant infection and 10.8% of those infected with the Delta variant in an observational study in the United Kingdom (UK) had long COVID.^47^ Despite the relatively low morbidity, the increased transmissibility of the Omicron variant may result in a larger number of potential patients with long COVID.^48^ Thus, it is essential to develop medications with special effects against long COVID to alleviate its clinical manifestations.

The approved vaccines have been proven to be highly effective in preventing COVID-19, especially severe illness.^49,50^ Notably, these vaccines also exhibit the capacity to prevent long COVID.^51^ In participants who received an mRNA or adenovirus vector COVID-19 vaccine, 12.8% and 8.8% decreases in long COVID morbidity were initially observed after one dose and two doses, respectively.^52^ Similarly, in a study involving 739 COVID-19 participants from Italy, the prevalence of long COVID was found to be 41.8% in unvaccinated individuals, 30.0% in those with one dose, 17.4% in those with two doses, and 16.0% in those with three doses, showing a correlation between the number of vaccine doses and long COVID prevalence.^53^ Compared to one dose, two vaccination doses also lower the risk of a larger range of manifestations, including myocarditis, myalgia, cerebral hemorrhage, anosmia, and interstitial lung disease within six months after infection, among other symptoms.^54^ In addition to reducing morbidity, a sentinel cohort study conducted in the United Kingdom emphasized the crucial role of vaccination in lowering the mortality rate among long COVID patients,^55^ further implying the importance of vaccination. However, the data from the Israeli nationwide health care organization indicated that, among the sequelae of long COVID, the administration of a vaccine led to merely a reduced prevalence of dyspnea in cases with a breakthrough infection.^43^ The differences in vaccines and races may be the underlying cause of the inconsistent results above. Despite the potential protective effect of vaccines, a higher antibody titer is correlated with worse sequelae,^56^ suggesting that an excessive immune response should be considered during vaccination.

Notably, increasing reinfection is extensively associated with the additional morbidity of long COVID sequelae involving cardiovascular, pulmonary, endocrine, hematological, gastrointestinal, mental, urinary, neurological, and musculoskeletal disorders.^57^ Therefore, adopting adequate strategies to prevent reinfection is necessary. Although vaccine administration holds a remarkably protective effect, the risk of long COVID remains high.^57,58^ Moreover, breakthrough SARS-CoV-2 infection (BTI), which refers to the occurrence of SARS-CoV-2 infection within 14 days of vaccination, increases the risk of experiencing various long COVID manifestations, including coagulation and hematologic, cardiovascular, kidney, gastrointestinal, neurologic, metabolic, and musculoskeletal disorders.^59^ Thus, reliance on vaccines is incapable of optimally mitigating the persistent sequelae of COVID-19, and prevention strategies for SARS-CoV-2 infection and treatment options for sequelae should be emphasized to enhance the quality of life of patients.

## Symptoms and possible mechanisms

### Neuropsychiatric system

The clinical manifestations of the neuropsychiatric system, which primarily include memory loss, sensorimotor aberration, cognitive disorder, paresthesia, loss of smell or taste, dizziness, and audiovestibular symptoms,^60–66^ seem to be the prominent features of long COVID, severely impeding the daily activities of patients. Among multiple neuropsychiatric symptoms, a meta-analysis indicated that fatigue and cognitive manifestations were the most prevalent symptoms of long COVID, with proportions of 32% and 22%, respectively.^67^ Cognitive disorder is a complicated neuropsychological syndrome characterized by the impairment of thinking, perceiving, and remembering.^68^ According to a recent study of 236,379 patients, the estimated incidences of parkinsonism, dementia, anxiety, and psychotic syndromes among cognitive disorders following six months of SARS-CoV-2 infection were 0.11%, 0.67%, 17.39%, and 1.40%, respectively.^69^ Within the field of cognitive symptoms, approximately a quarter of patients suffer from “brain fog”, a newly proposed syndrome encompassing attention deficits, processing speed issues, language fluency difficulties, memory problems, and executive function disorders.^70,71^ Even though these symptoms have low morbidity, cognitive impairments still require sufficient attention. A cohort study with a follow-up period up to 2 years after COVID-19 onset implied that common psychiatric manifestations such as mood disorders and anxiety returned to normal within 2 months, whereas the risks of brain fog, dementia, epilepsy, and psychotic disorders were still increasing at the end of 2 years.^72^ Additionally, sufficient emphasis should be placed on children due to the higher risk of persistent seizures/epilepsy and psychotic disorders compared with adults.^72^ Therefore, fully understanding the pathophysiological mechanisms of these manifestations is critical for prevention and treatment.

There are several possible hypotheses for their pathophysiology. The first explanation is the existence of an active-virus reservoir in the nervous system and neuronal injury.^73–75^ An in vitro experiment assessed SARS-CoV-2 infection in nerve cells and brain organoids and found that viral proteins and infectious particles of the virus were profiled in brain organoids.^73^ Simultaneously, cortical neurons and neural precursor cells were verified to be the direct binding site of SARS-CoV-2,^73^ suggesting that the virus probably invades the human brain and contributes to neuropsychiatric symptoms such as anosmia, ageusia, encephalitis, and Guillain‒Barre syndrome. Among nonhuman primates, sporadic SARS-CoV-2 has been detected in the brains of infected animals.^74^ Moreover, the pathophysiological processes of neuroinflammation, hypoxia, and microhemorrhages have also been observed,^74^ providing novel perspectives on the neuropsychiatric manifestations of long COVID. Aside from animal experiments, complete autopsies and sampling from the central nervous system of COVID-19 patients have shown that viral replication widely occurs in tissues/organs, including the brain, at 7 months after acute infection.^75^ In ORF6 and ORF10 of the SARS-CoV-2 proteome, two amyloidogenic subsequences with self-assembled structures were screened to have high toxicity for neurons by means of nanoscale imaging, spectroscopy, molecular modeling, kinetic assays, and X-ray scattering,^76^ triggering the neurologic symptoms of patients and further supporting the impact of persistent SARS-CoV-2 in neuropsychiatric symptoms of long COVID.

Neuroinflammation has also been confirmed as an imperative mechanism for the neuropathology of long COVID.^77^ An investigation based on the UK Biobank comprising 401 COVID-19 participants showed that a reduction in brain size as well as reduced gray matter thickness in the parahippocampal gyrus and orbitofrontal cortex were noted via magnetic resonance imaging (MRI) at 141 days after infection.^78^ Similar structural changes were also observed in a German study,^79^ indicating the possibility of neuroinflammatory events and degenerative hallmarks in patients with long COVID. To elucidate the underlying mechanisms, an in vivo experiment illustrated that inflammation-associated cytokines/chemokines such as CCL11 might promote hippocampal microglial activity and inhibit neurogenesis, which was comparable to the neuropathophysiology of cancer therapy and contributed to cognitive impairment.^80,81^ Inflammasome activation induced by viral infection also facilitates the activation of transforming growth factor beta (TGF-β) signaling as well as oxidative overload, thus resulting in Alzheimer’s disease-like features and cognitive disorders.^82^ Furthermore, a study on golden hamsters revealed immune cell activation and the production of proinflammatory cytokines in the olfactory bulb and olfactory epithelium, which even existed one month after SARS-CoV-2 clearance.^83^ These outcomes underscore the role of neuroinflammation in persistent neuropsychiatric symptoms.

Injury to blood vessels is likewise involved in the pathophysiology of the neuropsychiatric manifestations of long COVID. The impaired vessel density of retinal capillary microcirculation was shown to be more noticeable in long COVID patients than in controls,^84^ validating that blood vessel damage might promote persistent symptoms. Furthermore, endothelial dysfunction has been proven to facilitate long COVID. A retrospective study revealed that elevated levels of endothelial cell markers, such as von Willebrand factor (VWF) antigen and VWF propeptide, were observed in COVID-19 patients after an average of 68 days following infection,^85^ signifying sustained endothelial dysfunction in long COVID patients. Similarly, another study indicated that patients with COVID-19 developed endothelial dysfunction over 6 months after discharge compared with healthy participants.^86^ Nevertheless, the impact of endothelial dysfunction on cerebrovascular manifestations of long COVID merits further investigation.

### Myalgic encephalomyelitis/chronic fatigue syndrome

Myalgic encephalomyelitis/chronic fatigue syndrome (ME/CFS) is a neuroimmune illness characterized by intolerance to systemic exertion and chronic fatigue that cannot be alleviated via rest.^87,88^ The diagnostic criteria of ME/CFS comprise substantial impairment in daily activities for a minimum of 6 months and profound fatigue of new/definite onset that cannot be relieved by rest, accompanied by post-exertional malaise and unrefreshing sleep, cognitive impairment and/or orthostatic intolerance.^89^ For most patients with ME/CFS, ‘infectious-like’ manifestations involving fever, respiratory and digestive symptoms, myalgia, and lymphadenopathy universally emerge before illness onset.^60,87,89^

Fatigue syndromes after confirmed infection that might meet the diagnostic criteria of ME/CFS have been reported for multiple pathogens, including Ebola virus, Epstein-Barr virus, herpesvirus-6, SARS-CoV, and *Mycoplasma pneumoniae*.^90–94^ The potential correlation between long COVID and ME/CFS has also been commented by many researchers. A systematic review of 21 studies revealed that the clinical manifestations of ME/CFS exhibited many overlaps with long COVID.^95^ The presence of ME/CFS has also been observed in both children and adults with confirmed long COVID.^96,97^ Among participants over 18 years old with long COVID, 58.7% met the criteria for ME/CFS.^96^ In a cross-sectional survey, 40% of children and adolescents with COVID-19 were reported to have documented ME/CFS.^97^

The pathophysiological processes of ME/CFS could be summarized as initial immune and inflammatory responses, vascular dysregulation, and autonomic/metabolic adaptation.^89,98^ The widespread inflammatory response triggered by SARS-CoV-2 infection has been reported in patients with varying degrees of illness severity,^80,99^ which could occur in the nervous system and contribute to neuropsychiatric symptoms.^80,83^ The interaction networks of ME/CFS and long COVID, including inflammatory cytokines comprising interleukin (IL)-6 and IL-1B, common genes, and microRNAs, have recently been depicted,^100^ revealing the potential mechanism of ME/CFS in the occurrence of long COVID and further emphasizing the significance of the inflammatory response. Meanwhile, the dysregulation of endothelin-1 has been found in long COVID patients and cases presenting exertion intolerance and persistent fatigue, suggesting the emergence of endothelial dysfunction and its effect on ME/CFS.^101^ Moreover, metabolic disorders of energy probably promote exercise intolerance and nervous symptoms. The dysregulation of mitochondrial membrane potential and plasma metabolites related to mitochondria-dependent lipid catabolism are manifested in patients with long COVID,^102,103^ perhaps conducive to ME/CFS development. Intriguingly, a study implementing machine learning to assess antibody-binding data revealed that the microbiota-immune axis was involved in the pathophysiology of ME/CFS,^104^ suggesting that the microbiota could serve as a novel direction for investigating ME/CFS in post-COVID-19 syndromes.

Under certain circumstances, several diseases, such as mast cell activation syndrome, postural orthostatic tachycardia syndrome (POTS), intracranial hypertension, and craniocervical obstructions, are generally comorbid with ME/CFS.^105–107^ Quite a few studies have also documented the corresponding manifestations in patients with long COVID.^26,95^ However, the comorbid diseases of ME/CFS in long COVID remain enigmatic and worth further investigation.

### Cardiovascular system

Cardiovascular system symptoms remain one of the dominating persistent manifestations of long COVID. The prevalence of cardiac symptoms differs among current studies.^26,108–110^ In a study integrating data for individuals from 56 countries, the main symptoms of chest pain, palpitations, and fainting occurred in 86.04% of individuals with long COVID.^26^ For COVID-19 patients, a study of 153,760 patients showed that the burden of cardiovascular outcome per 1000 persons was 49.37 for dysrhythmia, 2.44 for inflammatory heart disease, and 18.47 for ischemic heart disease at 12 months after the COVID-19-positive test.^110^ Furthermore, POTS, a specific subtype of cardiovascular manifestation, was observed in approximately 30% of patients with.^111^

Several potential hypotheses of cardiovascular manifestations caused by long COVID have been proposed recently. Cardiac injury could occur in the acute or post-acute phase after viral infection. During the acute stage, the elevation of cardiac markers such as cardiac troponin signifies myocardial ischemia and injury.^112^ Regarding the mechanism, it has been confirmed that direct viral toxicity plays a crucial role in mediating myocardial injury by affecting angiotensin-converting enzyme 2 (ACE2) receptors.^113^ A pathological analysis of autopsy indicated that cardiac thrombi were observed in 78.6% of patients, and microthrombi in small muscular arteries, capillaries, and arterioles were noted in 64.3% of patients with myocyte necrosis.^114^ Thus, thrombosis is also an essential mechanism resulting in myocardial injury. The potential explanation of thrombosis is that SARS-CoV-2 directly targets pericytes with high ACE2 expression and consequently induces endothelial cell dysfunction,^115,116^ contributing to increased microvascular endothelial permeability and ultimately leading to thrombotic complications in microcirculation. Furthermore, the upregulation of cytokines IL-6, IL-1, and tumor necrosis factor (TNF)-α might also trigger endothelial dysfunction and platelet activation to facilitate thrombosis,^99^ which could eventually cause myocardial damage.

The mechanisms of persistent cardiac injury in the post-acute and chronic disease stages are still unclear. One possible mechanism is that the persistent presence of the virus may contribute to the development of cardiac symptoms in individuals with long COVID. Several studies have revealed that SARS-CoV-2 is detectable in plasma, stool, urine, and multiple organs for more than 4 months,^117–119^ causing persistent damage. Apart from viral persistence, autoantibodies also exert vital effects on cardiovascular system symptoms. Multiple studies have verified the high level of autoantibodies in patients with long COVID, such as antibodies against ACE2, β2-adrenoceptors, and M2 receptors.^60,117,120,121^ Hence, autoimmunity against cardiac antigens may cause chronic damage to the cardiovascular system.

### Respiratory system

Respiratory symptoms such as cough and dyspnea are common clinical manifestations of long COVID.^26,122–124^ An Italian study demonstrated that 43.4% of COVID-19 patients had persisting dyspnea at 60 days following onset.^122^ In addition, an international study reported that approximately 40% and 20% of patients with long COVID had dyspnea and dry cough over 6 months, respectively.^26^ This evidence shows that the respiratory manifestations of COVID-19 may persist for an extended duration and be worth heeding.

On account of acute respiratory disease, quite a number of COVID-19 patients, especially those with severe/critical disease, may suffer from diffuse alveolar damage, severe endothelial injury, and thrombosis,^125^ accordingly causing substantial and persistent injury to the lungs. Nevertheless, many patients with mild/moderate illness develop small airway dysfunction in the post-acute phase via quantitative chest computed tomography (CT) evaluation.^126^ A probable explanation is persistent SARS-CoV-2 in lung tissue.^119^ Thus, direct viral toxicity could constantly cause damage to the respiratory system. In some COVID-19 patients with ongoing dyspnea, increased expression levels of cytokines such as IL-1β, IL-6, and IL-8 have been observed,^99,127^ which may also contribute to pulmonary fibrosis. Furthermore, the dynamics of the airway immune microenvironment could induce respiratory manifestations. In assessments of the immune-proteomic landscape between healthy controls and long COVID patients, the expression levels of proteins correlated with apoptosis and epithelial injury have been shown to be higher in long COVID patients, and ongoing cytotoxic T-cell activation probably facilitated long-term lung injury.^128^ Likewise, another study revealed a positive correlation between SARS-CoV-2-specific T cells and systemic inflammation, while a negative correlation was observed with lung function,^129^ signifying that SARS-CoV-2-specific T cells may play an essential role in respiratory symptoms.

### Digestive system

The gastrointestinal symptoms of long COVID mainly include heartburn, gastrointestinal disorders, constipation, loss of appetite, and abdominal pain.^130–133^ An online survey of individuals from 56 countries showed that 20.5% of participants with long COVID experienced persistent diarrhea, and 13.7% of long COVID patients had loss of appetite, even 7 months after infection.^26^ A prospective study of 749 COVID-19 survivors revealed that gastrointestinal symptoms were experienced by approximately 29% of participants after a period of 6 months following viral infection.^134^ Among them, the leading digestive manifestations included reflux/heartburn (16.3% of cases), constipation (11.1% of cases), diarrhea (9.6% of cases), abdominal pain (9.4% of cases), and nausea/vomiting (7.1% of cases).^134^ Thus, the effect of digestive system manifestations on the quality of life of patients with long COVID cannot be neglected.

The probable hypotheses of the mechanisms regarding gastrointestinal symptoms comprise the persistent viral component or active virus and alterations in gut microbiota. Multiple studies have been undertaken to validate persistent SARS-CoV-2 in the gastrointestinal tract and found that viral RNAs and proteins in stool and gut tissue could be detected up to 12 months after diagnosis.^135–137^ By performing an endoscopy study, it was observed that a significant number of patients still had detectable levels of the nucleocapsid protein of SARS-CoV-2 in the gut epithelium even 7 months after the initial infection.^137^ Furthermore, most patients with antigen persistence were reported to have long COVID, which did not occur in the participants without persistent antigens.^137^

The gut microbiota is considered a complicated ecosystem that significantly affects human health and multiple diseases.^138^ Recent evidence has also revealed that the gut microbiota exerts a critical function in gastrointestinal symptoms of long COVID.^22,139–141^ The microbiome of long COVID patients is characterized by elevated levels of *Bacteroides vulgatus* and *Ruminococcus gnavus*, along with a decreased abundance of *Faecalibacterium prausnitzii*,^22^ which may facilitate the digestive manifestations of long COVID. Intriguingly, the gut microbiota has recently been validated to have predictive potential. Opportunistic pathogens of the gut are related to respiratory symptoms, while nosocomial pathogens such as *Actinomyces naeslundii* and *Clostridium innocuum* may contribute to neuropsychiatric manifestations, and butyrate-producing bacteria present an inverse association with long COVID.^22^ Analogously, patients with persistent reduction in pulmonary diffusing capacity for carbon monoxide have an altered gut microbiota composition characterized by the reduced abundance of dozens of bacterial taxa and increased levels of several taxa involving *Veillonella*.^142^ Therefore, the gut microbiome should serve as a promising research focus to elucidate the mechanisms of long COVID among multiple systems beyond the digestive system.

### Vascular and organ impairment

Despite initial recognition as a disease of the respiratory system, COVID-19 is capable of inducing multiple systemic and organic lesions. Damage to the circulatory system includes endothelial dysfunction, subsequent bleeding and thrombogenesis among long COVID patients. The level of arterial stiffening in the aorta, carotid artery, and brachial artery in COVID-19 patients was shown to be notably superior to that in the control group,^143^ indicating that the impairment of great vessels is a universal phenomenon in COVID-19. In regard to microcapillaries, participants with long COVID presented persistent microclots that were resistant to fibrinolysis,^144^ which exert a crucial role in blocking microcirculation. In addition, persistent capillary rarefication was found in patients at 18 months following SARS-CoV-2 infection,^25^ emphasizing the impaired microcirculation in individuals with long COVID. Vascular impairment at different sites might result in diverse manifestations. Neurovascular damage and endothelial cell activation, accompanied by subsequent platelet aggregation and microthrombi, have been reported in COVID-19 patients,^145^ which possibly causes sustained neuropsychiatric symptoms. With respect to the cardiovascular system, microthrombi are the major reason for myocardial damage in COVID patients.^114^ Furthermore, the risk of lung embolism, deep venous thromboembolism, and bleeding was still dramatically elevated after a long infection period compared to a control period,^146^ highlighting the role of vascular impairment in multisystem damage.

Recent evidence has focused on the multiorgan impairment of long COVID. Compared to controls, German patients who recovered from mild/moderate COVID-19 illness have been observed to have a higher risk of long-term sequelae, which includes multiorgan damage associated with renal, thrombotic, cardiac, and pulmonary functions.^147^ Furthermore, an observational cohort study noted that 70% of individuals with long COVID exhibited evidence of damage to at least one organ.^148^ Of them, the lungs (11%), heart (26%), kidneys (4%), pancreas (40%), liver (28%), and spleen (4%) suffered from mild impairment, with 29% of patients presenting with multiorgan damage.^148^ For certain organic impairments, the symptoms may be specific. Elevation of aminotransferases serves as the representative symptom of long COVID patients with liver impairment, which is perhaps caused by viral infection of the liver and supported by pathological manifestations.^149^ Analogously, SARS-CoV-2 has been observed to potentially have a direct impact on the pancreas by binding to ACE2 receptors and causing pancreatitis, diabetes, and pancreatic exocrine dysfunction.^150–152^ For kidney impairment, a study of 478 follow-up patients reported a reduced estimated glomerular filtration rate in 29.7% of patients who had no manifestations of acute kidney injury.^153^ However, the correlation between long COVID and chronic kidney disease is still obscure.^154^

### Reproductive system

Although studies exploring the potential correlation between long COVID and reproductive manifestations are relatively insufficient, reproductive symptoms have been frequently reported in individuals experiencing the post-acute syndrome of COVID-19. Menstrual alterations are the leading morbidity of reproductive symptoms in women with COVID-19. In a study involving 1031 women, 53% of participants experienced worse premenstrual symptoms, 18% had new onset of menorrhagia, and 30% had new dysmenorrhea after the COVID-19 pandemic.^155^ In the CoVHORT study, approximately 16% of COVID-19 participants had alterations in their menstrual cycle, which encompassed irregular menstruation, infrequent menstruation, and heightened premenstrual symptoms, persisting for a prolonged period.^156^ In addition, ovarian impairment, including reproductive endocrine disorder and decreased ovary reserve, was observed in COVID-19 patients.^157^ Mechanistically, follicular fluid from women who had previously been infected with SARS-CoV-2 exhibited decreased levels of IL-1β and vascular endothelial growth factor (VEGF), which subsequently influenced the expression of estrogen receptor β and the migration of endothelial cells,^158^ perhaps contributing to ovarian dysfunction. Nonetheless, another observational study held an inverse outcome that the function of the ovary was not adversely affected following SARS-CoV-2 infection, and the menstrual alterations might be attributed to inflammation or psychological factors.^159^ Thus, in-depth investigation with a larger sample size on the relationship between long COVID and ovarian impairment is necessary.

Regarding the male reproductive system, recent studies have exhibited the impairment of spermatogenic function and sperm quality in patients with COVID-19, which was potentially led by direct SARS-CoV-2 attack or immune activation.^160,161^ A large retrospective study compared the risk of many manifestations of long COVID in 486,149 cases who experienced the SARS-CoV-2 infection with 1,944,580 participants without evidence of infection and indicated that the morbidity of reproductive symptoms such as ejaculation difficulty and reduced libido was increased in COVID-19 patients.^162^ As another typical reproductive manifestation, erectile dysfunction was likewise documented in patients experiencing COVID-19.^163^ To elucidate its biological mechanism, transmission electron microscopy was conducted on penile tissue and revealed that persistent viral particles of SARS-CoV-2 were found to remain in the vascular endothelial cells of the penis, potentially playing a role in the development of erectile dysfunction.^163^

It is worth noting that patients with well-documented ME/CFS commonly have several reproductive manifestations, including menstrual cycle fluctuations, polycystic ovary syndrome (PCOS), and hyperprolactinemia.^164–166^ Because over 50% of patients with long COVID have the classic symptoms of ME/CFS,^96^ some of them probably also have reproductive comorbidities. Therefore, future studies should lay emphasis on the comorbidity with reproductive manifestations and ME/CFS in long COVID patients to better elucidate its pathophysiology.

### Musculoskeletal system

The musculoskeletal manifestations of long COVID, including musculoskeletal pain, sarcopenia and decreased skeletal muscle mass, have recently attracted much attention.^130,167–171^ The Linköping COVID-19 Study reported that 28.5% of individuals had weakness in the extremities, and 10.5% of individuals had muscle weakness among patients with long COVID symptoms.^172^ In addition, another study indicated that approximately 18.59% and 15.09% of survivors developed joint pain and myalgia at 6 months following hospitalization, respectively.^173^ In spite of the relatively low morbidity, these symptoms are classified into one of four major subphenotypes (nervous and musculoskeletal system manifestations) of long COVID and supported as core symptoms by 92% of patients with long COVID and their family members/caregivers,^169,174^ which signifies that delving deeply into their pathophysiological mechanisms might assist in the precise management of long COVID.

Similar to other systems, the direct attack of SARS-CoV-2 is considered a significant contributing factor to musculoskeletal system disorders. Multiple cellular types in skeletal muscle tissue, such as pericytes and smooth muscle cells, have been validated to express AEC2 and TMPRSS2 via analysis of transcriptional data,^175^ which indicates that SARS-CoV-2 has the potential to directly invade these cells, leading to detrimental effects on the musculoskeletal system. However, the autopsy studies found that the load of SARS-CoV-2 was low or even negative in tissue samples of COVID-19 patients who exhibited obvious musculoskeletal symptoms,^176–178^ demonstrating that the directly viral attack is difficult to comprehensively elucidate the musculoskeletal manifestations of long COVID. Moreover, inflammation and microvascular injury are also hypothesized to be crucial pathophysiological mechanisms. Systemic inflammation, characterized by persistently elevated levels of cytokines, including IFN-γ, TNF-α, IL-6, and IL-10, has been observed in numerous COVID-19 patients and could persist for over 6 months following SARS-CoV-2 infection.^179,180^ In this way, inflammatory cytokines potentially trigger abnormal catabolic pathways and result in muscle wasting.^181^ Interestingly, the studies of muscle biopsy showed that musculoskeletal injury ought to be the secondary outcome of microvascular damage, and substantial persistent circulating microclots with inflammatory molecules were profiled in patients with long COVID.^144,178^ Hence, the synergistic effect between inflammation and vascular injury could be an important element for maintaining musculoskeletal injury. Notably, other than catabolic pathways, inflammatory cytokines also promote the sensitization of the nervous system, offering a novel perspective for understanding the musculoskeletal symptoms of long COVID.^182^

Increasing evidence has pointed out the importance of hypoxia in skeletal muscle alterations.^167^ For patients with hypoxia, metabolic alterations and muscle wasting are common characteristics.^167^ Some patients with acute respiratory distress syndrome (ARDS) have been reported to develop skeletal muscle weakness and were unable to fully recover even after a 5-year follow-up after discharge.^183^ Intriguingly, a similar finding has been observed in hospitalized COVID-19 patients, especially those assigned to the ICU,^184,185^ which might be interpreted as hypoxia being widespread in patients with severe COVID-19 and probably facilitating skeletal muscle weakness. In mechanism, the hypoxia-induced factor (HIF) and related regulatory factors that stimulate hypoxia can delay muscle regeneration and cause the loss of skeletal muscle.^186^ Nevertheless, further elucidation is required to understand the underlying mechanisms of hypoxia in long COVID. Apart from the mechanisms above, several other factors, such as muscle disuse, malnutrition and medication, might also contribute to the reduced muscle mass and should be considered to explain the musculoskeletal manifestations of long COVID.

### Immune system

While the complete understanding of the immunobiology of long COVID remains elusive, the possible mechanisms in multiple systems have underscored its importance. The dominant hypotheses include persistent SARS-CoV-2 and corresponding antigens and/or nucleic acids involved in the inflammatory response, persistent autoantibodies triggering autoimmunity during the post-acute stage, and an imbalance in the microbiome and immune microenvironment.^60,136,187–189^ Several studies have confirmed the presence of SARS-CoV-2 RNAs and proteins in the cardiovascular system, brain, lung tissue, plasma, and urine for a long time after initial infection,^60,117–119^ which probably results in chronic inflammation and multiple systemic manifestations. In a study of 37 long COVID participants, the circulating spike emerged in more than 70% of patients with ongoing cardiovascular, gastrointestinal, neuropsychiatric, systemic, and musculoskeletal symptoms,^136^ supporting the explanation that a reservoir of active SARS-CoV-2 or its components persists in COVID-19 patients. Nonetheless, the limitation of sample size makes it difficult to ensure reliability, and future research with larger sample sizes is necessitated to be conducted.

Autoimmunity plays a vital role in the development of long COVID. Multiple recent studies have documented the upregulation of autoantibodies such as antibodies to ACE2, anti-nuclear autoantibodies, and immunomodulatory factors (including complement components, chemokines, cytokines, and cell-surface proteins) in patients with persistent symptoms following SARS-CoV-2 infection.^120,121,190–192^ An observational study indicated that the upregulation of IgG antibodies against ACE2 was found in 1.5% of patients with COVID-19,^191^ implying the widespread existence of autoantibodies to ACE2. However, the duration of increased levels of anti-ACE2 antibodies and their relationship with long COVID still require further elucidation. In terms of anti-nuclear autoantibodies, the expression of anti-nuclear/extractable-nuclear antibodies (ANAs/ENAs) was higher in COVID-19 patients at 6 months after recovery than in healthy participants or patients with other respiratory infections, and persistent positive titers were relatively associated with the severity of clinical manifestations,^192^ implying that anti-ANA/ENA antibodies could facilitate long COVID. Furthermore, the elevated level of specific autoantibodies against immunomodulatory factors has been reported to contribute to particular immune-cell population depletion and worse clinical outcomes,^121^ and multiomics data analysis of 309 COVID-19 patients revealed cross-correlations between neutralizing antibodies against SARS-CoV-2 and autoantibodies.^120^ For instance, there was a negative correlation between the expression of anti-nuclear and anti-IFN-α2 autoantibodies and the levels of anti-SARS-CoV-2 IgG,^120^ suggesting a potential interconnection between these factors and the development of long COVID. Intriguingly, several autoantibodies have been found to be correlated with disorders of specific organs. For instance, long COVID patients with neurological manifestations show a higher level of nucleocapsid protein IgG against SARS-CoV-2, while some other autoantibodies have been associated with sputum production and gastrointestinal symptoms,^120^ manifesting their potential as diagnostic biomarkers.

The dynamics in the immune microenvironment could also be associated with long COVID.^193^ For patients who go on to develop long COVID, TNF-α and IFN-γ-induced protein 10 are significantly elevated during early recovery (<90 days), while IL-6 is upregulated among patients with long COVID,^194^ demonstrating that persistent immune activation potentially leads to long COVID and could be utilized to develop novel remedies. An in-depth study indicated that long COVID patients had a typical immune microenvironment featuring activated innate immunocytes, poor abundance of naïve lymphocytes (T and B cells), and overexpression of IFN-β and IFN-λ1 at 8 months following infection.^195^ Furthermore, the patterns of plasma-cell-related expression and monocyte alterations at the acute stage of COVID-19 were also correlated with persistent symptoms,^196,197^ providing early insights into the etiologies and pathophysiology of long COVID. However, there remain numerous unknown fields concerning the immune microenvironment of long COVID, and further investigation may shed light on its prevention and treatment.

### Psychological disorders and long COVID

Although most studies on long COVID are predominantly concerned with somatic manifestations and enrich the understanding of its pathophysiology to a certain extent,^7,42,60^ the effect of psychological and psychosomatic factors has been almost neglected. In fact, psychological disorders may affect a substantial proportion of those who develop long COVID.^198–205^ A previous study reported that both COVID-19 patients and control individuals develop clinical manifestations consistent with PASC.^198^ Despite the relatively superior morbidity among COVID-19 patients, no evidence of autoimmunity, abnormal immune activity or persistent infection was found in the entire study population.^198^ Hence, the underlying contributing factors rather than the somatic factors mentioned above deserve to be underlined. Significantly, individuals with perceived fatigue in the long COVID group did not exhibit any additional objective fatigability compared to those without long COVID symptoms,^199^ signifying that mental factors probably serve as critical modifiers or even causes of long COVID. Analogously, another study showed that 64% of patients after mild COVID-19 with persistent neurological symptoms met the criteria of somatic symptom disorder (SSD),^200^ and psychiatric conditions were confirmed to contribute to the prolonged duration of COVID-19 symptoms,^206^ which suggests that psychosomatic disorders should be emphasized when investigating the mechanisms of long COVID. Consequently, the identification of psychological or psychosomatic disorders is encouraged to assist in multidisciplinary care and better cure long COVID.^201,207^

With the accumulation of clinical evidence, the mechanisms of psychological/psychosomatic disorders in long COVID have also been highlighted. Psychiatric consequences have been proven to affect the immune system via multiple pathways, such as the hypothalamic-pituitary adrenal (HPA) axis and microglial activation, which subsequently might delay the recovery of COVID-19.^208,209^ In addition, somatic symptoms have been verified to lead to worse mental health and poor quality of life, while mental disorders could aggravate the physical symptoms of long COVID patients in turn.^134,210^ Under these circumstances, the positive feedback loop between somatic symptoms and psychological disorders probably exacerbates both mental and physical manifestations, which further facilitates long COVID.^134^ However, although the importance of psychological disorders has been underlined, the mechanisms are still unclear. There are several reasons why psychological disorders in long COVID have not yet been well studied. First, it is difficult for clinicians to investigate the underlying pathology by ascribing psychological disorders.^211^ Second, the explanations of physical symptoms from the perspective of psychology are usually considered a stigma for patients.^212^ Third, the roles of psychosomatic medicine in other diseases have not yet been clarified,^213^ making it more difficult to elucidate the progression of long COVID. Therefore, thoroughly illustrating the psychological mechanisms of long COVID remains an enormous challenge. Notably, a cross-sectional analysis of 26,823 participants suggested that self-reported SARS-CoV-2 infection was correlated with the presence of long COVID symptoms, while seropositivity was merely associated with anosmia instead of other physical symptoms.^202^ Thus, it is speculated that persistent somatic symptoms may be related more to self-reported infection than to laboratory-confirmed SARS-CoV-2 infection. Consequently, misdiagnosis might occur, and patients with a diagnosis of long COVID are prone to experiencing multilayered stigmas, further exacerbating their mental symptoms.^214^ Therefore, a careful evaluation is indispensable to prevent the manifestations caused by psychological disorders being incorrectly ascribed to PASC or long COVID.

Despite the huge challenge of elucidating its psychological mechanisms, it is assured that psychological and psychosomatic factors exert an indispensable role in the progression of long COVID manifestations, and adopting multidisciplinary therapeutic strategies, including psychotherapy, is potentially beneficial for persistent symptoms.

## Management of long COVID

### Diagnostic tools for long COVID

For early detection of long COVID, routine auxiliary inspections such as medical imaging techniques and laboratory examinations are currently indispensable. Among them, echocardiography, cardiac biomarkers and MRI are utilized to assess cardiac injury and arrhythmia,^215^ and chest imaging examination and pulmonary function tests are adopted for evaluating lung damage and the degree of dyspnea.^216^ However, for certain neuropsychiatric manifestations, such as fatigue and cognitive impairment, the evidence is mainly based on self-report of patients rather than objective tests.^26,217^ Although (18)F-FDG PET has a satisfactory performance in predicting hyposmia, cognitive impairment, and insomnia via the identification of bilateral hypometabolism of the bilateral rectal or orbital gyrus in previous research,^218^ no apparent change in glucose metabolism of the brain has been observed in another study.^219^ Therefore, it is necessary to develop more accurate laboratory tests to enhance the sensitivity and specificity for predicting long COVID.

In addition to traditional examination methods, the microbiome has recently been reported to be associated with long COVID.^104,220–222^ Early research into the gut microbiome suggested that opportunistic gut pathogens were correlated with persistent respiratory manifestations. The common nosocomial pathogens, including *Actinomyces naeslundii* and *Clostridium innocuum*, were related to fatigue and neuropsychiatric symptoms (*p* < 0.05).^22^ Simultaneously, multikingdom microbiota identified by metagenomic-based clustering exhibit potential utility in long COVID prediction.^141^ Moreover, the alteration of tens of bacterial taxa, including *Veillonella*, has been validated to be related to fibrosis and could be utilized to diagnose the long-term pulmonary manifestations of long COVID.^142^ In a similar manner, the abundance of certain oral microbiota, including members of the *Veillonella* and genera *Prevotella*, is significantly increased in long COVID patients, which exhibits the potential for predicting persistent symptoms after SARS-CoV-2 infection.^222^ The available evidence indicates that the composition of the microbiome can be potentially used to predict the occurrence of long COVID, even specific symptoms.

Liquid biopsy research into the clinical manifestations of long COVID has shown satisfactory prognostic and diagnostic potential.^223–232^ For example, kynurenine has been validated to be upregulated in the serum and saliva of long COVID-19 patients over 20 weeks,^223^ and vascular transformation biomarkers, including P-SEL and ANG-1, have excellent specificity and sensitivity for long COVID and may serve as promising biomarkers for long COVID diagnosis and monitoring.^228^ In addition, several biomarkers have the capacity to predict neuropsychiatric manifestations that are difficult to detect in traditional inspection. Neurofilament light chain, glial fibrillary acidic protein, SARS-CoV-2 nucleocapsid antigen, and immune-inflammation indicators in plasma have been verified to be correlated with persistent depression/anxiety and cognitive impairment.^225,227^ The neurofilament light and 14-3-3 protein from cerebrospinal fluid were significantly related to neurologic disability at 18 months after neurologic symptom onset.^224^ Remarkably, extracellular vesicles derived from neurocytes contain the crucial components particularly relevant to occult neural damage and indicate the capacity for monitoring the neuropsychiatric manifestations of long COVID.^229,231^

The relevance of noncoding RNAs to many human diseases, such as malignant tumors, cardiovascular disorders, and infectious diseases, including COVID-19, has been well studied.^233,234^ In a pilot study, miR-29a-3p, miR-155-5p and miR-146a-3p exhibited excellent performance in COVID-19 diagnosis, and miR-29a-3p and miR-146a-3p could be utilized to distinguish the post-acute phase of SARS-CoV-2 infection from the acute phase.^235^ Hence, noncoding RNAs are also hypothesized to possess the potential to detect and monitor long COVID.

Although the diagnostic performance of liquid biopsy for long COVID has been observed, most of the evidence is based on preliminary studies with limited sample sizes. Therefore, the importance of exploring biomarkers suitable for clinical practice cannot be overemphasized.

### Remedies for long COVID

**Fig. 4:**
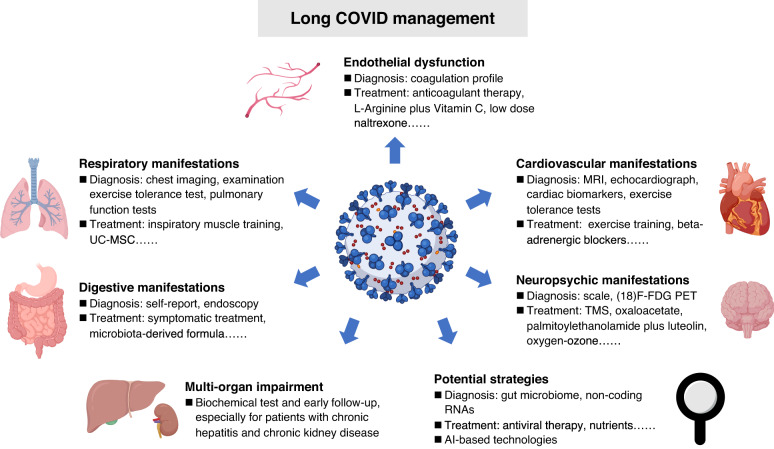
Multidisciplinary management of long COVID. Multidisciplinary management including diagnostic tools and treatment options based on previous experience and pilot studies is essential for recovery of long COVID patients. AI artificial intelligence, MRI magnetic resonance imaging, TMS transcranial magnetic stimulation, UC-MSC umbilical cord-derived mesenchymal stem cell

To help clinicians better manage long COVID manifestations, the National Institute for Health and Care Excellence (NICE) of England developed guidelines on the care of patients with persistent effects of COVID-19.^9,236^ Although there is no documented evidence regarding effective remedies for patients with long COVID, abundant therapeutic regimens based on previous experiences for certain symptoms and pilot studies have been proposed as the means to effectively address the post-acute symptoms of COVID-19^9,237^ (Table 1) (Fig. 4).

**Table 1:** Major candidate treatments for long COVID

Manifestations	Treatment options	Supporting evidence	references
Respiratory symptoms	Self-management including stopping smoking and regular exercise	COPD and long COVID literatures	^239,240^
	Inspiratory muscle training	Long COVID RCT	^241^
	Music-based approaches	Long COVID RCT	^242^
	UC-MSC treatment	COVID-19 RCT	^243^
Cardiovascular symptoms	β-adrenergic blockers	Heart disease literature	^245^
POTS	Non-pharmacological interventions: health education and exercise training Pharmacological treatments: β-blockers and vasoactive agents	POTS literature	^246^
Chronic fatigue	Self-management	NICE guideline for long COVID	^236^
ME/CFS	Energy management, personalized exercise or physical activity, personalized sleep management, and dietary management	ME/CFS literature	^247^
	Anhydrous enol-oxaloacetate	Long COVID pilot study	^248^
	Hyperbaric oxygen therapy	Long COVID pilot study	^249^
	Oxygen-ozone autohemotherapy	Long COVID pilot study	^250^
Digestive symptoms	SIM01 (a microbiota-derived formula)	COVID-19 pilot study	^252^
Endothelial dysfunction	L-Arginine plus Vitamin C	Long COVID pilot study	^253,254^
	Low dose naltrexone	COVID-19 pilot study	^255^
MASC	H1 and H2 antihistamines	MASC literature	^256^
Olfactory disorders	Nasal irrigation (including ambroxol, betamethasone, and rinazine)	Long COVID pilot study	^258^
Memory and olfactory dysfunction	Palmitoylethanolamide plus luteolin	Long COVID pilot study	^259^
Viral persistence	Nirmatrelvir	Long COVID pilot study	^265^
Long COVID	Nutrients treatment	Long COVID pilot study	^263,264^

*COPD* chronic obstructive pulmonary disease, *MCAS* mast cell activation syndrome, *ME/CFS* myalgic encephalomyelitis/chronic fatigue syndrome, *NICE* National Institute for Health and Care Excellence, *POTS* postural orthostatic tachycardia syndrome, *RCT* randomized controlled trial, *UC-MSC* umbilical cord-derived mesenchymal stem cell

Respiratory symptoms are frequently reported among individuals with long COVID, which mainly manifest as persistent dyspnea and cough.^26^ The exercise tolerance test and chest radiograph are recommended in the NICE and ESCMID guidelines to assist in illness evaluation and management.^9,236,238^ To better control pulmonary manifestations, multidisciplinary approaches should be applied. The recommendations for dyspnea suggest that self-management, including avoiding pollutants, stopping smoking, and regular exercise, could relieve exacerbated dyspnea.^42,239,240^ For long COVID patients, previous studies showed that inspiratory muscle training and music-based breathing training elicited clinical improvements in chest symptoms and difficult breathing,^241,242^ which should be recommended for COVID-19 patients. Additional therapeutic regimens proposed for managing the respiratory symptoms of long COVID based on pilot studies also facilitate symptomatic relief. Human umbilical cord-derived mesenchymal stem cell (UC-MSC) administration for patients with COVID-19 has revealed an excellent outcome in symptoms and lung lesion improvement at 1 year after infection compared to a control group.^243^ Furthermore, the COVID-Rehab study proposed a cardiopulmonary rehabilitation program to treat individuals with long COVID.^244^

For cardiovascular symptoms, the treatment options for other cardiovascular disorders/syndromes can be used for reference in long COVID. In accordance with the NICE guideline, exercise tolerance tests should be applied routinely for heart function measurement.^42,236^ β-adrenergic blockers have been amply validated to benefit patients with cardiac arrhythmias, acute coronary syndromes, and angina^42,245^; thus, it could be implied that β-adrenergic blockers might possess potential efficacy in the treatment of cardiovascular symptoms associated with long COVID. As a special type of cardiovascular symptom, POTS featured as orthostatic intolerance and the excessive increase of heart rate while standing is commonly reported in long COVID.^26,246^ The treatment of POTS mainly consists of nonpharmacological interventions such as health education and exercise training as well as pharmacological treatments including β-blockers and vasoactive agents.^246^ Therefore, rational usage of these approaches is perhaps beneficial for long COVID patients. However, few studies have explored the therapeutic effects of these interventions in long COVID.

Chronic fatigue holds the dominant place in morbidity of long COVID, which even accounts for 80% of patients with long COVID.^26^ Because of the lack of specific treatment, the NICE guideline initially recommended self-management for relieving fatigue.^236^ Nevertheless, patients with long COVID fatigue are commonly comorbid with ME/CFS; thus, active therapeutic strategies are necessary. Multidisciplinary strategies have also been proposed to manage ME/CFS in current guidelines, which mainly involve energy management, personalized exercise or physical activity, personalized sleep management, and dietary management.^247^ Hence, these strategies are likely to be effective for managing the ME/CFS symptoms of long COVID. Nevertheless, it should be noted that ME/CFS manifestations cannot currently be cured, and the strategies mentioned merely control symptoms.^247^ Furthermore, many studies have investigated certain remedies in long COVID patients. A clinical trial evaluated the therapeutic effect of anhydrous enol-oxaloacetate and found that it significantly reduced the fatigue of ME/CFS in long COVID patients.^248^ However, the data for the control group is from a historical trial and meta-analysis, and further study of oxaloacetate with a rigorous design is warranted. In addition, oxygen-ozone autohemotherapy has exerted the ability to alleviate fatigue and pain in at least 67% of COVID patients, and hyperbaric oxygen therapy with 10 sessions yielded a substantial improvement in fatigue, cognition, executive function, attention, verbal function, and information processing.^249,250^ In general, these remedies documented in pilot studies demonstrate a favorable effect on ME/CFS of long-COVID and deserve in-depth study.

Moreover, multiple additional interventions have been applied to tackle the gastrointestinal, circulatory, neurologic, musculoskeletal and even multiorgan manifestations of long COVID. As a research hotspot of the digestive system, the gut microbiome has long been considered a potential direction for the treatment of gastrointestinal diseases. A previous study proved that 5-hydroxytryptamine signaling mediated by gut microbiome dysregulation probably contributed to the gastrointestinal manifestations of long COVID.^251^ Thus, certain agents targeting the gut microbiome may alleviate post-COVID manifestations. In this manner, SIM01, a microbiota-derived formula including xylooligosaccharide, galactooligosaccharides, resistant dextrin, and Bifidobacteria strains, relieved the gut dysbiosis and symptoms of COVID-19 patients,^252^ further validating the effect of the gut microbiome on treating digestive manifestations. Endothelial dysfunction and persistent plasma microclots have been found in the circulatory system, which potentially lead to damage to multiple organs, including the brain, lung, and heart.^144,253^ Therefore, active anticoagulant therapy is essential for COVID-19 patients in a hypercoagulable state to reduce the risk of thrombogenesis. For oxidation reduction and endothelial function improvement, the administration of L-arginine plus vitamin C for COVID-19 patients was applied in the LINCOLN study and significantly reduced the incidence of long COVID symptoms such as asthenia, dyspnea, chest tightness, dizziness, headache, and concentration difficulty.^253,254^ In the meanwhile, low-dose naltrexone exhibited a promising effect on reducing the incidence of thrombotic complications.^255^ Interestingly, naltrexone has also been mentioned in the treatment of ME/CFS.^60^ The evidence above demonstrates the potential of naltrexone for long COVID treatment. H1 and H2 antihistamines are commonly recommended for mast cell activation syndrome to mitigate the symptoms, but cognitive decline caused by H1 blockers should be noted.^256^ Various strategies have been proposed for the treatment of neuropsychiatric manifestations. For instance, obvious improvement in fatigue, cognitive function, and depressive symptoms has been observed after transcranial magnetic stimulation (TMS) in long COVID patients.^257^ With regard to persistent olfactory disorders, a recent study indicated that the combination of nasal irrigation (with ambroxol, betamethasone, and rinazine) and systemic prednisone represented an excellent curative effect to improve dysosmia in long COVID patients,^258^ and palmitoylethanolamide plus luteolin also ameliorated neuropsychiatric manifestations, including memory and olfactory dysfunction.^259^ For musculoskeletal pain, regular exercise was suggested to reduce pain and improve physical function.^260^ It is remarkable that these physical manifestations are perhaps aggravated by psychological symptoms; thus, psychotherapy interwoven with therapy of somatic consequences is of utmost importance to long COVID treatment.^261,262^

In addition to the L-arginine plus vitamin C mentioned above, nutrients, TGF-β inhibitors, and antiviral therapies exhibit a potential effect on alleviating multisystem or multiorgan symptoms.^254,263–266^ The proper intake of nutrients is strongly recommended to maintain metabolism and overcome disease during the COVID-19 pandemic.^264^ Among nutrients, *Morinda citrifolia* and fermented *Carica papaya* are hypothesized to diminish the manifestations of long COVID via redox balancing, pro-energy, and immune-modulating mechanisms.^263^ Regrettably, clinical outcomes are still lacking, so research concerning these nutrients in long COVID therapy is warranted. On account of persistent SARS-CoV-2 in multiple organs,^42,60^ antiviral therapies perhaps exert promising effects on long COVID symptom relief. With respect to antiviral agents, nirmatrelvir dramatically mitigates the progression of COVID-19 without additional safety concerns.^267^ Furthermore, patients administered nirmatrelvir presented a 26% reduction in morbidity associated with long COVID symptoms of fatigue, heart disease, blood clots, dyspnea, and cognitive impairment.^265^ Thus, nirmatrelvir should be routinely recommended to COVID-19 patients. TGF-β inhibitors, modulators of immunity and fibrosis, have also demonstrated favorable potential in attenuating long-term COVID symptoms.^266^ Nevertheless, their curative effects need further validation.

Overall, current studies have presented a diverse array of therapeutic options for combating long COVID. Nevertheless, the majority of them are based on previous experience in similar diseases and pilot studies with crude designs. Although hundreds of clinical trials have been registered (Table 2), few of them have been widely used in clinical practice. Accordingly, there is an urgent need for well-designed trials with large sample sizes to investigate the potential impact of updated therapeutic regimens, including nutrients, antiviral agents, and anticoagulants, on addressing the challenges of long COVID.

**Table 2:** Representative clinical trials for treatment of long COVID

Therapeutic regimens	NCT identifier	Indications	Phase	Developer
Nirmatrelvir-Ritonavir	NCT05668091 NCT05576662	Multiple symptoms	II	Harlan M Krumholz, Stanford University, Pfizer
Lithium	NCT05618587	Fatigue and brain fog	II	State University of New York at Buffalo
Sodium pyruvate nasal spray	NCT04871815	Multiple symptoms	II, III	Cellular Sciences, etc.
Mitoquinone	NCT05373043	Vascular endothelial dysfunction	NA	VA Office of Research and Development
Imatinib-Infliximab	NCT05220280	Multiple symptoms	IV	Clinical Urology and Epidemiology Working Group, etc.
TNX-102 SL	NCT05472090	Pain	II	Tonix Pharmaceuticals
Remdesivir	NCT04978259	Multiple symptoms	IV	Clinical Urology and Epidemiology Working Group, etc.
Nicotinamide adenine dinucleotide plus naltrexone	NCT04604704	Fatigue	II	AgelessRx
UC-MSC-derived exosomes	NCT05808400	Chronic cough	I	Huazhong University of Science and Technology, etc.
Acupuncture	NCT05212688	Fatigue	II	Royal Marsden NHS Foundation Trust
Sirolimus	NCT04948203	Pulmonary fibrosis	II, III	University of Chicago
Prospekta	NCT05074888	Fatigue	III	Materia Medica Holding
Ivabradine	NCT05481177	POTS	IV	Uniformed Services University of the Health Sciences
Temelimab	NCT05497089	Neuropsychiatric symptoms	II	GeNeuro SA
Metoprolol Succinate	NCT05096884	Tachycardia, Dyspnea	I	Hackensack Meridian Health
Vortioxetine	NCT05047952	Cognitive impairment	II	Brain and Cognition Discovery Foundation
Mind body syndrome therapy	NCT04854772	Somatic symptoms	NA	Beth Israel Deaconess Medical Center
Microbiome immunity formula	NCT04950803	Multiple symptoms	NA	Chinese University of Hong Kong

*NA* not applicable, *POTS* postural orthostatic tachycardia syndrome, *UC-MSC* umbilical cord-derived mesenchymal stem cell

### Artificial intelligence (AI) in long COVID management

In the past few decades, AI technologies have developed rapidly and paved the way for precise diagnosis and clinical decision-making for multiple diseases, including malignant tumors, respiratory diseases, and pediatric diseases, on the basis of medical image and electronic health record (EHR) data.^268–270^ In the field of COVID-19, state-of-the-art AI models have also been applied for rapid diagnosis and severe illness prediction.^271,272^ The above studies demonstrate that AI has the ability to tackle complex clinical tasks and empower personalized medicine.

In spite of the relatively young discipline, cutting-edge AI technologies have been utilized in the management of patients with long COVID. On the one hand, AI technologies enable the accurate identification of long COVID. Liquid biopsy is deemed a potent tool for disease diagnosis and monitoring. A recent study conducted a high-definition single-cell assay to compare long COVID patients with normal donors and identified specific cellular/acellular events of long COVID.^273^ Using these events, a machine learning classifier was developed to separate long COVID patients from healthy controls, with an accuracy over 90%.^273^ Apart from blood examination, constructing models based on EHR data could also enable accurate diagnosis.^109,274,275^ The XGBoost models trained with diagnostic information, health-care utilization, demographics, and medications attained areas under the curve (AUCs) of 0.92 (whole patients), 0.85 (non-hospitalized individuals), and 0.90 (hospitalized individuals) for long COVID identification.^275^ Interestingly, the symptoms in the acute period after SARS-CoV-2 infection were verified to be related to long COVID,^109,276^ and a random forest model created using the manifestations at 7 days plus personal characteristics and comorbidities had the capacity to predict individuals with persistent symptoms,^109^ which offers a powerful tool for the early identification of individuals with a high risk of long COVID. Moreover, a multiclass machine-learning model based on fecal metagenomic data was also reported to hold promising potential in diagnosing PACS.^221^

On the other hand, AI technologies have been utilized for the stratification of long COVID patients. Researchers have recently proposed an unsupervised machine learning model for semantic phenotypic clustering utilizing EHR data that divides long COVID patients into 6 subtypes, including multisystem symptoms plus laboratory abnormalities (Cluster 1), pulmonary disorders (Cluster 2), neuropsychiatric manifestations (Cluster 3), cardiovascular manifestations (Cluster 4), pain/fatigue (Cluster 5), and multisystem-pain symptoms (Cluster 6).^277^ Consequently, this method enables the rational allocation of clinical resources and precision clinical management. Nevertheless, another study based on machine learning analysis suggested that long COVID patients should be classified into 4 subphenotypes consisting of renal and cardiac phenotype; respiratory, anxiety and sleep phenotype; musculoskeletal and nervous phenotype; and respiratory and digestive phenotype.^169^ These differences may be derived from the data bias, selection of cluster number, and variety of machine learning models. Thus, a large sample set from multiple centers is essential for precise subdivision of long COVID phenotypes. Moreover, the instruments for measuring the symptom burden of long COVID individuals have also exhibited the capacity for assessing the effect of interventions and enable precise clinical management.^278–280^ However, they are presented in the form of questionnaires, and AI-based patterns should be constructed.

**Fig. 5:**
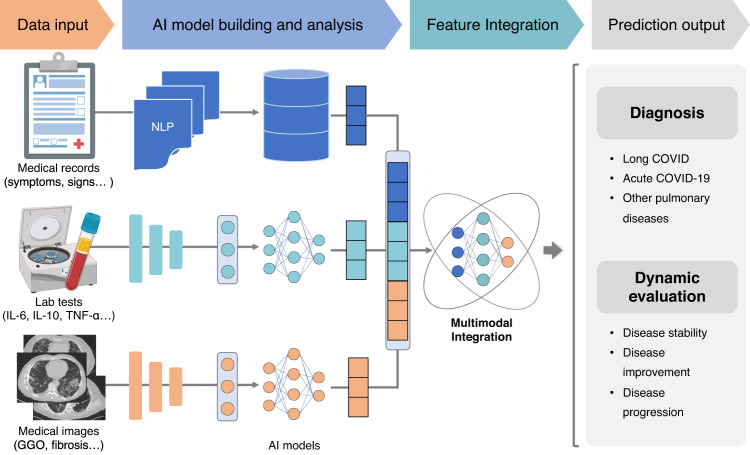
The workflow of multimodal integration (MMI) for precise management of long COVID. MMI systems could identify associations among multimodal data and output the outcomes including diagnosis and dynamic evaluation of patients to empower the precise management. AI artificial intelligence, GGO ground-glass opacity, NLP natural language processing

As an essential instrument for the diagnosis and prognosis evaluation of pulmonary diseases, CT examination also exhibits the potential for long COVID assessment.^281–286^ The CT abnormalities among patients with persistent manifestations following acute COVID-19 manifest as subpleural bands and ground-glass opacity (GGO) at 3 months after infection as well as fibrosis without obvious GGOs at 6 months after infection.^283,285^ Even worse, fibrotic changes could persist for 1 year in some individuals.^281^ Thus, the early identification and dynamic evaluation of CT abnormalities is of paramount importance. Although qualitative CT approaches have been applied for assessment of the sequential change in long-term pulmonary manifestations of long COVID, they mostly rely on manual assessment.^282,284,287^ Therefore, establishing AI-based models to automatically assess the CT images of long COVID patients is extremely meaningful to improve the efficiency of doctors. Moreover, the current AI models for long COVID management are concentrated on single modality, which inevitably diminishes the synergistic effect among multiple clinical modalities. Indeed, multimodal integration (MMI) via AI has been substantiated to enhance the robustness and accuracy of models,^288–290^ lighting the path to clinical practice of AI production. Hence, further studies harnessing MMI technologies to integrate EHR, laboratory examination, and medical images may enable the individualized management of patients with long COVID (Fig. 5).

### Conclusions and future directions

The ongoing symptoms and post-acute sequelae of patients with SARS-CoV-2 infection have been increasingly brought to the forefront, which could contribute to multi-system manifestations encompassing cardiovascular, respiratory, neuropsychiatric, gastrointestinal, reproductive, and musculoskeletal symptoms. Moreover, advances in diagnosis and remedies for long COVID have been witnessed recently. Nevertheless, many problems left over from the past still require enough attention and urgently need to be addressed.

The first is the accessibility and accuracy of laboratory tests for SARS-CoV-2. The data from a previous study suggested that only 1–3% of patients had laboratory-confirmed COVID-19 in the first wave due to technical limitations.^291^ For non-hospitalized individuals with mild to moderate symptoms of COVID-19, PCR or antigen testing might not be applied.^60^ In addition, despite being the gold standard for diagnosis, the omission diagnostic rate of nucleic acid detection is still worth noting. Approximately 38% of cases underwent an omission diagnosis at symptom onset, and the false-negative rate was still 2% on days 22–24.^292,293^ However, the majority of patients with nonsevere illness recovered from COVID-19 at that time, possibly resulting in missed diagnosis of numerous long COVID patients. In consideration of the accessibility of self-tests, antigen tests have also been recommended. However, only 64% of confirmed COVID-19 individuals experienced seroconversion, implying a considerably high proportion of omission diagnosis via antigen testing of COVID-19.^294^ The prevalence of nonseroconversion is more remarkable in mild COVID-19 patients and children.^295,296^ Among the diverse severity spectrum, the antigen levels of 22.2% mild and 2.6% severe patients with COVID-19 could not be profiled even after an 8-month follow-up.^295^ To improve the detection rate, cases with COVID-19-related manifestations should receive timely and sustained inspection. Since the combination of IgG and IgM showed promising sensitivity of up to 96%,^297^ the combination of IgG/IgM, antigen and PCR tests can be assumed to be a precise approach for COVID-19 diagnosis. By these means, long COVID may be better monitored.

In addition, a lack of knowledge and health-care education for sequelae of COVID-19 probably impedes personalized management. For instance, ME/CFS and POTS are common long COVID sequelae that have rarely been studied previously. Only 5.6% of US medical schools cover the clinical, curriculum, and research criteria for ME/CFS,^298^ suggesting that the proportion might be even lower in developing countries. Furthermore, a cross-sectional survey reported that 75% of POTS cases were misdiagnosed due to the complexity of the illness.^299^ Therefore, health facilities, medical schools, and government agencies should educate research/health workers on these sequelae and pursue the establishment of a dedicated discipline focused on long COVID research and care to enable the precise management of long COVID manifestations. Moreover, as recommended by researchers from Johns Hopkins University, the establishment of COVID-19 clinics equipped with multidisciplinary specialists is also crucial to afford integrated care for long COVID symptoms.^300^ Nonetheless, multidisciplinary clinics in low-/middle-income countries remain challenging due to limited resource availability and medical professionals.^301^

Despite the rapid progress in epidemiological understanding, pathophysiological mechanism and management strategies of long COVID, the available evidence is principally based on pilot studies with finite sample sizes and treatment of similar diseases.^60,302,303^ To address these problems, future research concerning the critical clinical, epidemiological, serological, and pathological characteristics will assist in understanding the pathophysiology of long COVID. Ultimately, it is imperative that a diverse range of long COVID patients of various races and ethnicities should be meaningfully engaged in clinical trials to facilitate the clinical applications of novel interventions.

There are several strengths in this review. First, we comprehensively summarize the epidemiological understanding, impact of vaccinations and variants, multiorgan manifestations and corresponding pathophysiological mechanisms, and multidisciplinary management of long COVID. Second, as abnormal laboratory examination results were not found in many patients and a considerable percentage of patients suffering from long COVID symptoms meet the criteria for SSD,^198,200^ the correlation between psychological or psychosomatic factors and long COVID manifestations is underlined in this review. Third, the major challenges concerning biological knowledge gaps and efficient remedies that need to be addressed are also proposed to move the research field of long COVID forward. Despite the advantages above, weaknesses still exist. Multiple therapeutic strategies have presented promising effects on alleviating symptoms of long COVID, but most of them might be overly optimistic because the current studies are mainly based on small sample sizes without rigorous scientific validation. In fact, these measures may bear a risk of serious side effects without medical advice. Hence, correct guidance is necessary to prevent patients with long COVID from being misled by social media platforms. Furthermore, among patients infected with the Omicron variant and/or vaccinated, the morbidity associated with long COVID is noticeably lower, and most of them are resolved in several months.^43,45^ Thus, some findings may currently be overrated. However, as the transmissibility of the Omicron variant increases,^48,304^ a larger proportion of patients are likely to develop long COVID. Consequently, sufficient attention still needs to be paid to the pathophysiological mechanisms and therapeutic options of long COVID.

Given the multisystemic illness of long COVID, numerous clinical manifestations, including impairment of multiple organs/systems, vascular damage, dysautonomia, and ME/CFS, have been observed in an extensive number of patients. Simultaneously, the foreseeable requirement of health care for sequelae of COVID-19 will continue to grow due to the global pandemic. However, the diagnostic and treatment strategies remain inadequate currently. To address these challenges, a combination of existing laboratory tests, suitable health education, outpatient infrastructure, and clinical trials with rigorous designs are clearly warranted.
